# Supplementary material for: Patterns of Intron Gain and Loss in Fungi
Source: PLoS Biol. 2004 Nov 30;2(12):e422. doi: 10.1371/journal.pbio.0020422 (PMC532390; doi:10.1371/journal.pbio.0020422)
Supplement: Table S1 — Also available at http://genes.mit.edu/NielsenEtAl/. (4.3 MB ZIP). [file pbio.0020422.st001.zip › NielsenEtAl/html/656.html]

AN2150.1.NCU04449.1.MG10357.1.FG05249.1


```
 CLUSTAL W (1.82) Multiple Sequence Alignments - Introns Inserted


Sequence 1: NCU04449.1	558 aa
Sequence 2: FG05249.1	553 aa
Sequence 3: MG10357.1	566 aa
Sequence 4: AN2150.1	594 aa
Alignment Length: 598 aa
Number Identitical Residues: 290 aa
Alignment Score (without introns) 14806


MG10357.1 	MEENKAKVQAQPAAEQSTEQP--------AEPSKSALKKAAKASAKEASKAVKASKAAP-
NCU04449.1	MEEN-------------------------KDQSKSALKKAEKAAQMAAKKAEKAAKQATL
FG05249.1 	MEGA-------------------------AEQSKSALKKAEKQARHAADKAAKAAKQTNL
AN2150.1  	MASQAPADNASVPQELPDRTQNPAGEAPQAEVSKNAAKKAAKQAEMAAKKAEKAAKAGKS
          	*    .  .:. . . .    ..:..:.. : **.* *** * :   *.** **:*    

MG10357.1 	-------VAVKKTD---DIIGITVSKEENFPQWYQEVVLKAEMIEYYTEISGFYILR~PA
NCU04449.1	-----PVVGGKKAD---DIIGITVKKSENFSQWYQEVVLKAELIEYYTEISGFFIMR~PA
FG05249.1 	-----PVVGGKKAD---DIIGITVSKADNFPQWYQEVVLKAEMVEYYTEISGFFVLR~PL
AN2150.1  	EKGQKPAGKGPKANPDKALIGIDVGKDVDFPGWYQQVLTKGDMLDYY-DVSGCYILK0PA
          	......     *::... :*** * *  :*. ***:*: *.::::** ::** :::: * 

MG10357.1 	AMFVWSQIRKWFQEKIDVMGVEEASFPMFLSSKSLEKEKDHVEGFAPELAWVTKA2GDKD
NCU04449.1	TMYIWNVIRKWFQEHIDELGVEETNFPMFLSQKSLEKEKEHVEGFAPELAWVTKA~GDKD
FG05249.1 	SMHIWNEIRKWFQGHIEEMGVDETNFPMFLSSKSLEKEKDHVEGFAPELAWVTKA2GDKD
AN2150.1  	SYSIWEEIQQWFNQRIKKIGVKNCSFPLFVSEDVLQREKDHIEGFAAEVAWVTHA2GSTP
          	:  :*. *::**: :*. :**.: .**:*:*.. *::**:*:****.*:****:* *.. 

MG10357.1 	LEVPVAVRPTSEAVMYPY~---YSKWIRSHRDLPLRLNQWNSVVRWEAKQTTPFLRAREF
NCU04449.1	LEVPVAVRPTSEAVMYPW1KPDYAKWIRSHRDLPFRLNQWNSVVRWEAKQTTPFLRAREF
FG05249.1 	LEVPVAVRPTSEAVMYPY~---YSKWIRSHRDLPLRLNQWNSVVRWEAKQTTPFLRTREF
AN2150.1  	LEKKIAIRPTSETVMYPY~---YAKWIRSHRDLPLKLNQWNSVVRWEFKHPQPFLRTREF
          	**  :*:*****:****:    *:**********::*********** *:. ****:***

MG10357.1 	LWQEGHTAHLTEEDAGKEVLEILEHYAGVYEQLLAVPVVRGKKTENEKFAGGYYTTTVEG
NCU04449.1	MWQEGHTAHLTEQAAGEEVLQILEFYAGVYEQLLAVPVVRGRKTEAEKFAGGYYTTTVEG
FG05249.1 	LWQEGHTAHLTEELAGAEVLEILELYAGVYEKLLAVPVVRGRKTENEKFAGGYYTTTVEG
AN2150.1  	LWQEGHTAHLTKEAAHEEVMYILDLYAQIYEELLAVPVVKGQKTEKEKFAGGLYTTTVEG
          	:**********:: *  **: **: ** :**:*******:*:*** ****** *******

MG10357.1 	YIPSNGRGIQGATSHCLGQNFSKMFDITVEDPKKEKG----HLHVWQNSWGLSTRVIGVM
NCU04449.1	YIPSNGRGIQGATSHCLGQNFSKMFDITVEDPAEKGK----HIHVWQNSWGLSTRVIGVM
FG05249.1 	YIPSNGRGIQGATSHALGQNFSKMFDITVEDPQKQGS----HIHVWQNSWGLSTRVIGVM
AN2150.1  	YIPATGRGIQGGTSHGLGQNFSKMFNITVEDPSAKGDEKKPPLHVWQNSWGLSTRTLGVM
          	***:.******.*** *********:******  :  .... :************.:***

MG10357.1 	VMIHGDDKGLVFPPRIARTQAVLIPVGITAKTTPEERTAHTAKLDELNKTLKDAGVRVES
NCU04449.1	VMIHGDDKGLVLPPRIAKIQSILIPVGLTAKMSAEEKDAHMKKVDELLATLKKAGVRADV
FG05249.1 	VMIHGDDKGLVLPPRVAKTQVVIVPVGINKKTTPEEKKKHEDQVEEIRANLKKAGVRVTA
AN2150.1  	VMIHSDDNGLVLPPRVAENQVVVVPVGITAKLTEEDRAKLYAEVDKITETLTAAGVRAIS
          	****.**:***:***:*. * :::***:. * : *::     :::::  .*. ****.  

MG10357.1 	DMRDGYTPAWKFNHWELKGVPLRLEFGPKDAAKDQVAFARRDTGEK---GSIAIADLATK
NCU04449.1	DTREGYTPAWKFNDWELKGVPLRIEFGPKDAAKDVVSYARRDTGEK---GTIPIAELTTK
FG05249.1 	DWREGYTPAWKFNDWELKGVPLRVEFGPKDAAKGVISTARRDTGEK---GEIAIADVATK
AN2150.1  	DKREGYSPGWKFNEWELRGVPLRIEFGPGESAGNFVSTARRDIPGKDGKGTIPIPELSTA
          	* *:**:*.****.***:*****:**** ::* . :: ****   *...* *.*.:::* 

MG10357.1 	VPELLETIQKAMYDKADAAFRSHRIVVAEWEKVVPALDSKNVVLIPHCLDGKCEDHIKDI
NCU04449.1	VPELLETIQQDMYNKAEVSFREHRLKIEKWDDVVPALDAKNVVLIPHCLVPECEDKIKEL
FG05249.1 	VPELLETIQSDMYNKAEKSFREHRLQITKWEDVLPALDNKNVVLIPFCGAPKCEDRIKEL
AN2150.1  	VPALLDTIHKDMFKRADDQYRTHRKLITNWDDFTPALNDKNICIIPHCLTEECEDQIKEM
          	** **:**:. *:.:*:  :* **  : :*:.. ***: **: :**.*   :***:**::

MG10357.1 	TKG--TGMELQADGQKAPSMGMKSLCIPFEQPEGLVEG-TKCLSPDCDKLAQKYVMFGR~
NCU04449.1	TTGRSDEAAEGPESQKAPSMGMKSLCIPFEQPEGLVKGETKCLNPECSRLAEQWCMFGR~
FG05249.1 	TTR--EEQPNVPDNEKLPTMGMKSLCVPFEQPEGIAAGETKCLNPECEGKADKWTMFGR~
AN2150.1  	SARKAEEDSGVAQDSRAPSMGAKSLCIPFDQPEGIVPGETKCTNPKCTRFAEKWCMFGR1
          	:   :      .:..: *:** ****:**:****:. *.*** .*.*   *::: **** 

MG10357.1 	SY
NCU04449.1	SY
FG05249.1 	SY
AN2150.1  	SY
          	**
```
